# Supplementary material for: Identification of Photocatalytic Alkaloids from Coptidis Rhizome by an Offline HPLC/CC/SCD Approach
Source: Molecules. 2022 Sep 21;27(19):6179. doi: 10.3390/molecules27196179 (PMC9570981; doi:10.3390/molecules27196179)
Supplement: Supplementary file 1 [file molecules-27-06179-s001.zip › molecules-1929855-supplementary.pdf]

Supporting Information

Identification of photocatalytic alkaloids from Coptidis rhizome by HPLC-CC-SCD approach

Shu-Qin Qin [1], Jun Ma [2], Qi-Qi Wang [1], Wei Xu [1], Wen-Cai Ye [1,*] and Ren-Wang Jiang [1,*]

[1] Guangdong Province Key Laboratory of Pharmacodynamic Constituents of TCM and New Drugs Research, College of Pharmacy, Jinan University, Guangzhou 510632.

[2] Research Institute of Guangdong HAID Group Co., Ltd., Guangzhou 511400, P. R. China.

Correspondence: trwjiang@jnu.edu.cn (Ren-Wang Jiang); chywc@aliyun.com (Wen-Cai Ye). Tel: 8620-85221016; Fax: 8620-85221559

Table of Contents Graphic

1. Materials

2. Extraction of total alkaloids from CR

3. Synthesis and identification of benzimidazole using CR as catalyst

Figure Sa. ^{1}H-NMR spectra of compound **1c** (400 MHz, DMSO).

Figure Sb. ^{13}C-NMR spectra of compound **1c** (100 MHz, DMSO).

Figure Sc. ESI-MS spectra of compound **1c** ($[M+H]^+$, observed m/z: 195.0911, calcd. m/z: 195.0916)

4. Figures for the NMR and crystal structures of NDS⊃(1-7)

Figure S1a NOESY spectra of **NDS⊃1**$_2$ (600 MHz, DMSO) showing the correlation between Hc of NDS and H-9 of **1**.

Figure S1b C–H···O interactions in (NDS)⊃**1**$_2$

Figure S1c C–H···π interactions in (NDS)⊃**1**$_2$

Figure S2a ^{1}H-NMR of **2** (I), **NDS⊃2**$_2$ (II) and NDS (III) (600 MHz, DMSO)

Figure S2b NOESY spectra of **NDS⊃2**$_2$ (600 MHz, DMSO) showing the correlation between Hc of NDS and H-15 of **2**.

Figure S2c C–H···O interactions in (NDS) ⊃**2**$_2$

Figure S2d C–H···π interactions in (NDS) ⊃**2**$_2$

Figure S3a. ^{1}H-NMR of **3** (I), **(NDS)**$_{1/2}$**⊃3** (II) and NDS (III) (600 MHz, DMSO)

Figure S3b. NOESY spectra of **(NDS)**$_{1/2}$**⊃3** (600 MHz, DMSO) showing the correlations between Hc of NDS and H-3 of **3**, and between Hc of NDS and H-15 of **3**

Figure S3c C–H···O interactions in (NDS)$_{1/2}$⊃**3**

Figure S3d C–H···π interactions in (NDS)$_{1/2}$⊃**3**

Figure S4a ^{1}H-NMR of **4** (I), **(NDS)**$_{1/2}$**⊃4** (II) and NDS (III) (600 MHz, DMSO)

Figure S4b NOESY spectra of **(NDS)**$_{1/2}$**⊃4** (600 MHz, DMSO) showing the correlation between Hc of NDS and H-2 of **4**.

Figure S4c C–H···O interactions in (NDS)$_{1/2}$⊃**4**

Figure S4d C–H···π interactions in (NDS)$_{1/2}$⊃**4**

Figure S5a ^{1}H-NMR of **5** (I), **NDS⊃5**$_2$ (II) and NDS (III) (600 MHz, DMSO)

Figure S5b NOESY spectra of **NDS⊃5**$_2$ (600 MHz, DMSO) showing the correlation between Ha of NDS and H-5 of **5**.

Figure S5c C–H···O interactions in (NDS) $\supset\mathbf{5}_2$

Figure S5d C–H···π interactions in (NDS) $\supset\mathbf{5}_2$

Figure S6a. ^{1}H-NMR of **6** (I), **(NDS)**$_{1/2}\supset$**6** (II) and NDS (III) (600 MHz, DMSO)

Figure S6b. NOESY spectra of **(NDS)** $_{1/2}\supset$**6** (600 MHz, DMSO) showing the correlations between Hc of NDS and H-8, H-15 of **6**, and between Hb of NDS and H-15 of **6**.

Figure S6c C–H···O interactions in (NDS)$_{1/2}\supset$**6**

Figure S6d C–H···π interactions in (NDS)$_{1/2}\supset$**6**

Figure S7a. ^{1}H-NMR of **7** (I), **(NDS)** $_{1/2}\supset$**7** (II) and NDS (III) (600 MHz, DMSO)

Figure S7b. NOESY spectra of **(NDS)** $_{1/2}\supset$**7** (600 MHz, DMSO) showing the correlation between Hb of NDS and H-5 of **7**.

Figure S7c C–H···O interactions in (NDS)$_{1/2}\supset$**7**;

Figure S7d C–H···π interactions in (NDS)$_{1/2}\supset$**7**;

5. Co-crystallization conditions

6. NDS decrease the required amounts of alkaloids to nanoscale

Figure S8 Nanoscale crystallization of NDS and **3**(jatrorrhizine).

7. Interaction geometries in the co-crystal complexes

8. Single-crystal X-ray diffraction analysis

9. Electrostatic potential surfaces

1. Materials

The herbal materials of Coptidis rhizome were purchased in a shop in Guangdong Province, China in 2021. The herb was identified by Prof. Guang-Xiong Zhou (Jinan University, China). A voucher specimen (CR-001) has been deposited with the College of Pharmacy, Jinan University. The NMR spectra were obtained on Bruker AV-400 spectrometer with chemical shift reported in δ (ppm) using TMS as the internal stand. X-ray diffraction of compound was collected on a Rigaku Oxford diffractometer. Analytical HPLC was performed on an Agilent 1200 system. Preparative HPLC was carried out on Wufeng LC-100 system. Chromatographic grade solvents were used for all HPLC analysis. All other reagents are analytically pure, and used without further purification.

2. Extraction of total alkaloids from CR

Commercially purchased CR herb was crushed and sieved to obtain CR powder. The powder (1g) was extracted by ultrasonic with 95% EtOH at room temperature for three times. The solution was filtered and concentrated under reduced pressure to obtain the crude extract. Then, appropriate amount of 2% HCl solution was added to solve the crude extract and adjust the pH= 4. The solution was filtered and the filtrate was alkalized with NaOH to pH= 9, which was then extracted with dichloromethane. Finally, the solvent was removed under reduced pressure to afford the total alkaloids of CR (80 mg).

3. Synthesis of benzimidazole using CR as catalyst

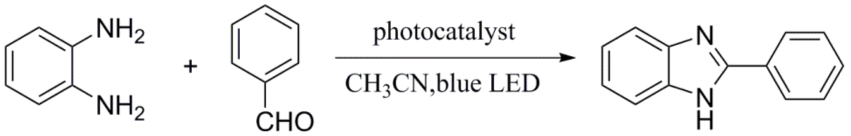

1a 1b 1c

Benzaldehyde (0.1 mmol), *o*-phenylenediamine (0.1 mmol), acetonitrile (1.5 mL) and catalyst (3 mol%) were added in a tube (10 ml). The open-air reaction tube was placed under a 5 W blue LED lamp and stirred at room temperature for 12 hours. The reaction was extracted with ethyl acetate, and the organic layer was washed with water. The organic layer was saturated with sodium chloride and separated. Then the organic layer was dried with anhydrous Na_2SO_4 and concentrated under reduced pressure. The crude product was purified through the silica gel column chromatography (petroleum ether: ethyl acetate = 2:1) to afford a light-yellow product (**1c**). ^{1}H NMR (400 MHz, DMSO) δ 12.93 (s, 1H), 8.23 – 8.15 (m, 2H), 7.65 – 7.44 (m, 5H), 7.25 – 7.15 (m, 2H). ^{13}C NMR (100 MHz, DMSO) δ 151.21, 130.13, 129.85, 128.95, 126.45, 122.12, 115.13.

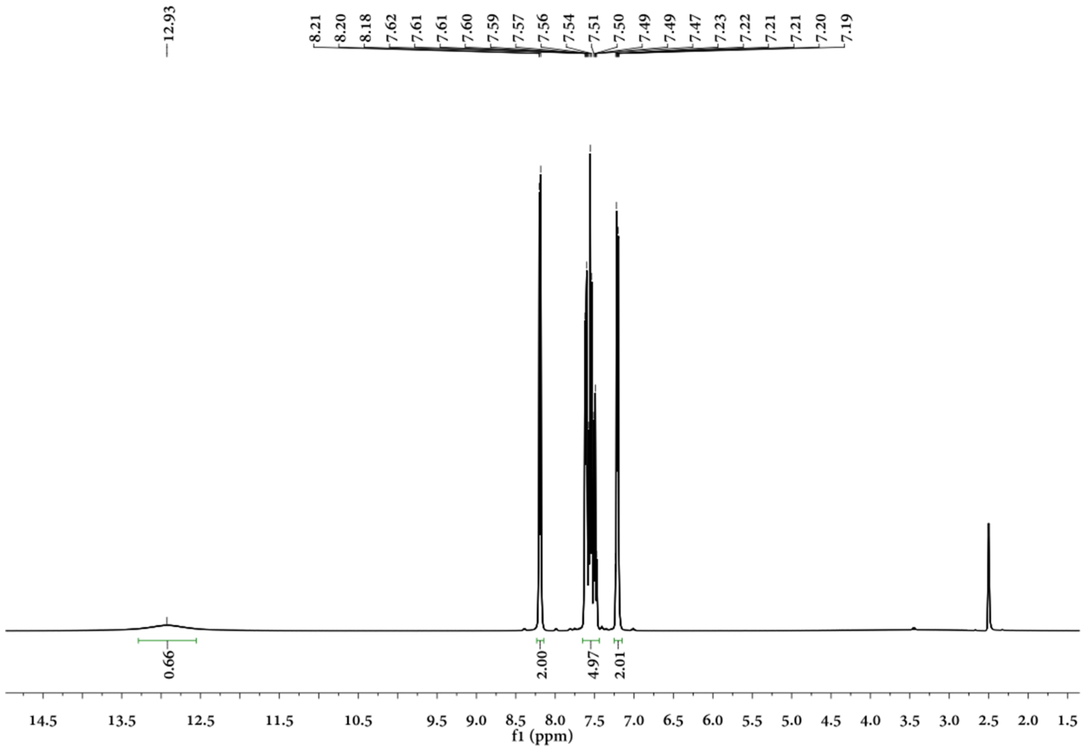

Figure Sa. ^{1}H-NMR spectra of compound **1c** (400 MHz, DMSO).

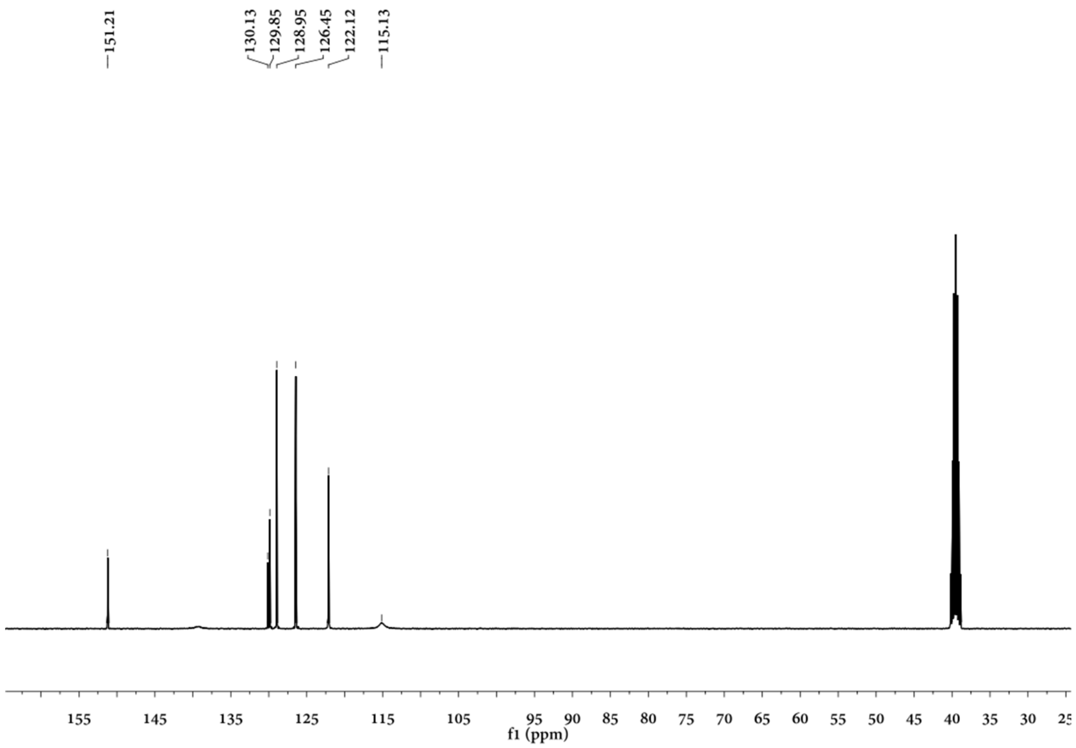

Figure Sb. ^{13}C-NMR spectra of compound **1c** (100 MHz, DMSO).

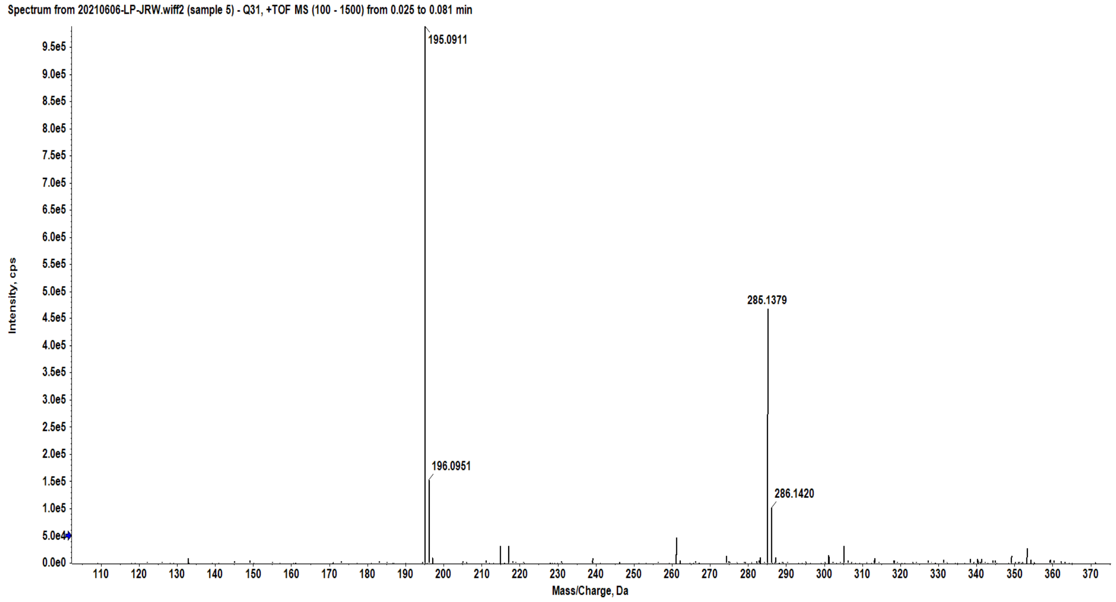

Figure Sc. ESI-MS spectra of compound **1c** ($[M+H]^+$, observed m/z: 195.0911, calcd. m/z: 195.0916)

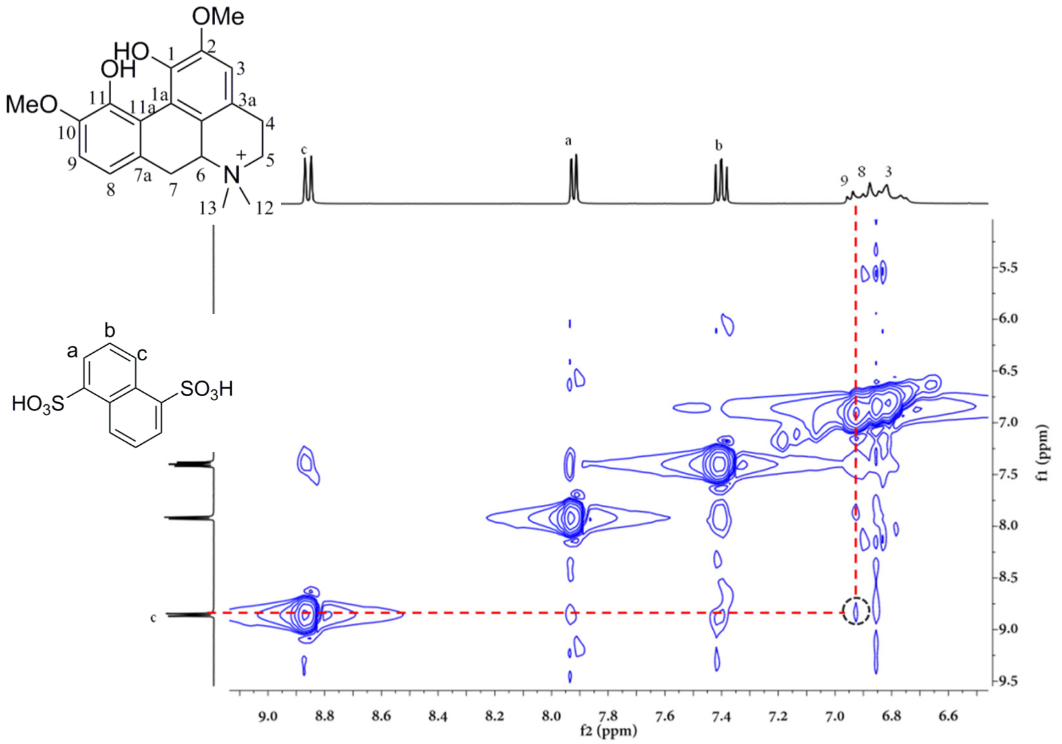

Figure S1a NOESY spectra of **NDS⊃1**$_2$ (600 MHz, DMSO) showing the correlation between Hc of NDS and H-9 of **1**.

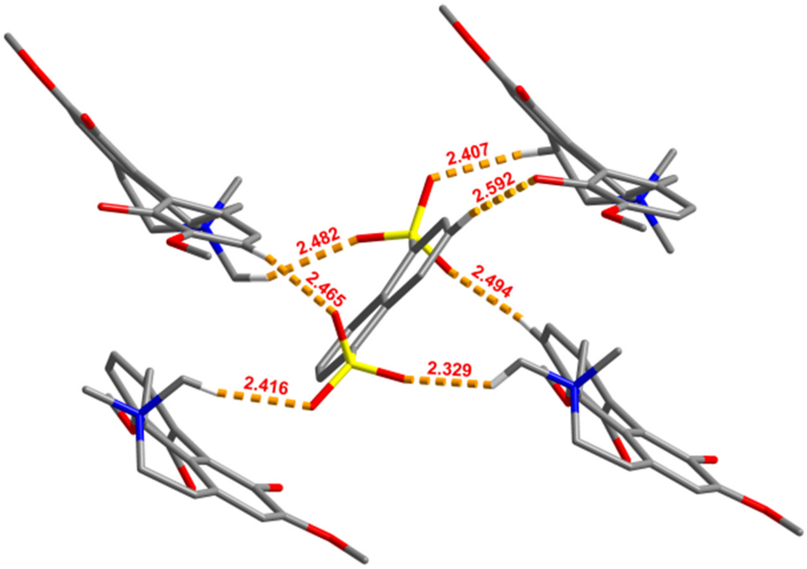

Figure S1b C–H···O interactions in **NDS⊃1**$_2$

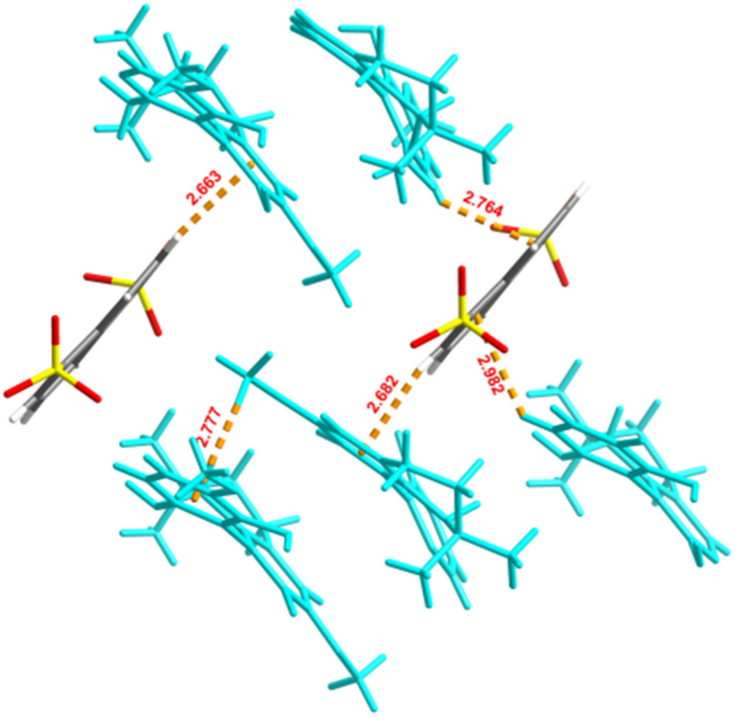

Figure S1c C–H···π interactions in **NDS⊃1₂**

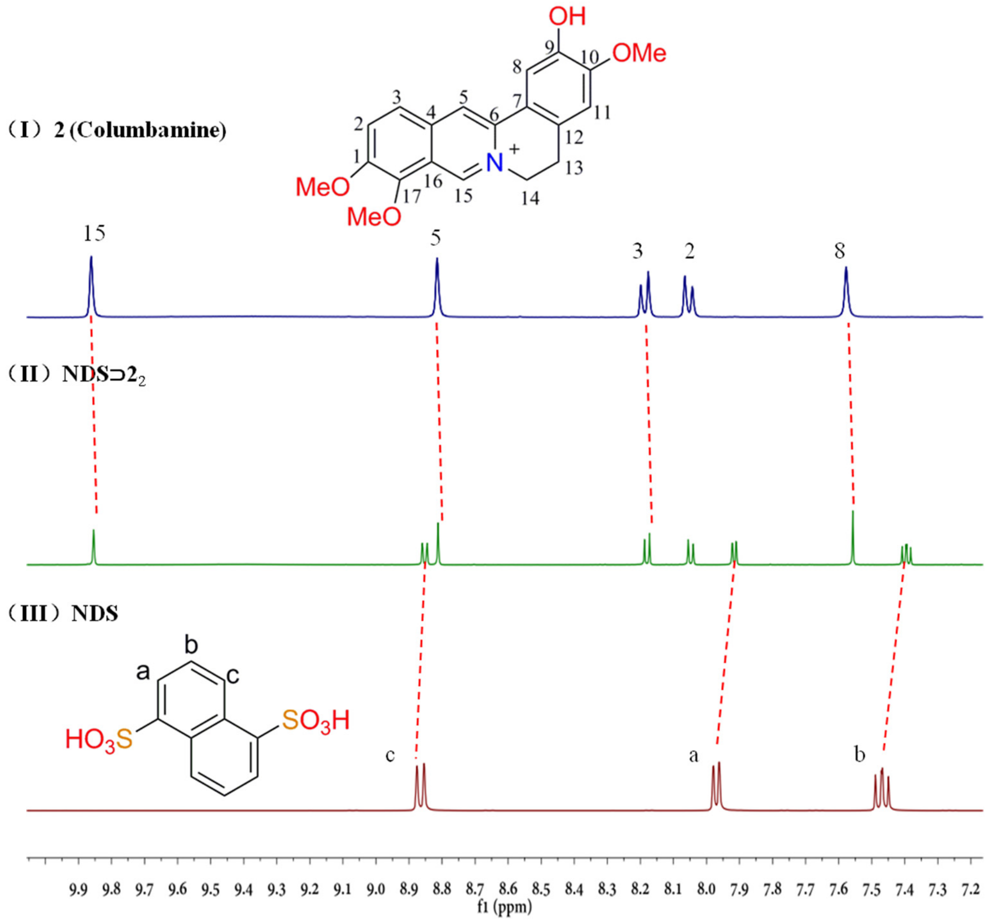

Figure S2a ^{1}H-NMR of **2** (I), **NDS⊃2**$_2$ (II) and NDS (III) (600 MHz, DMSO)

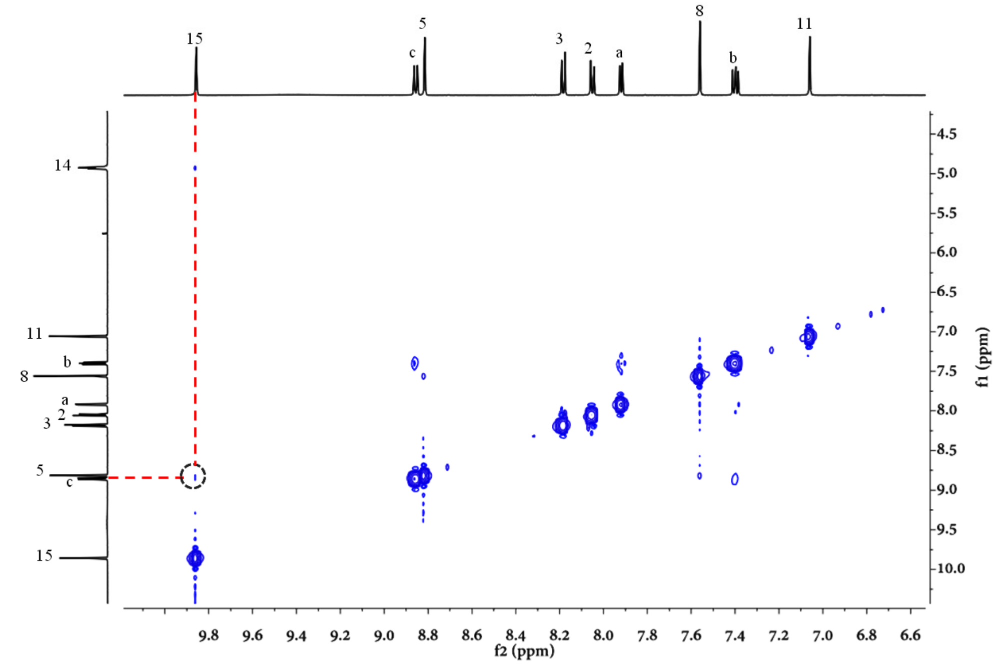

Figure S2b NOESY spectra of **NDS⊃2**$_2$ (600 MHz, DMSO) showing the correlation between Hc of NDS and H-15 of **2**.

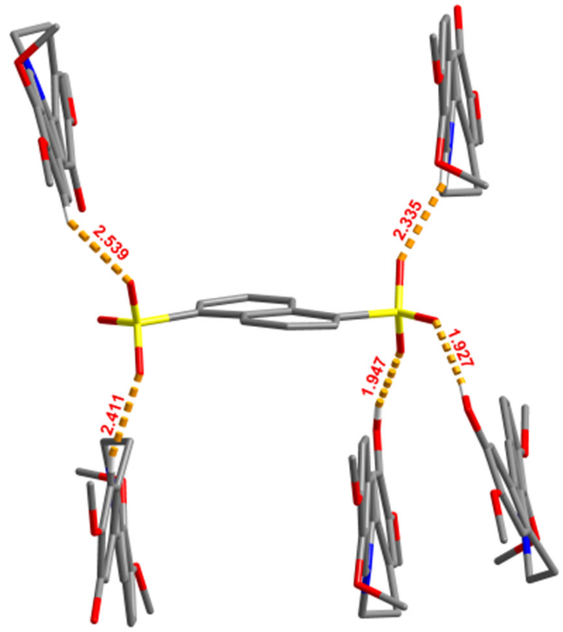

Figure S2c C–H···O interactions in **NDS⊃2**$_2$

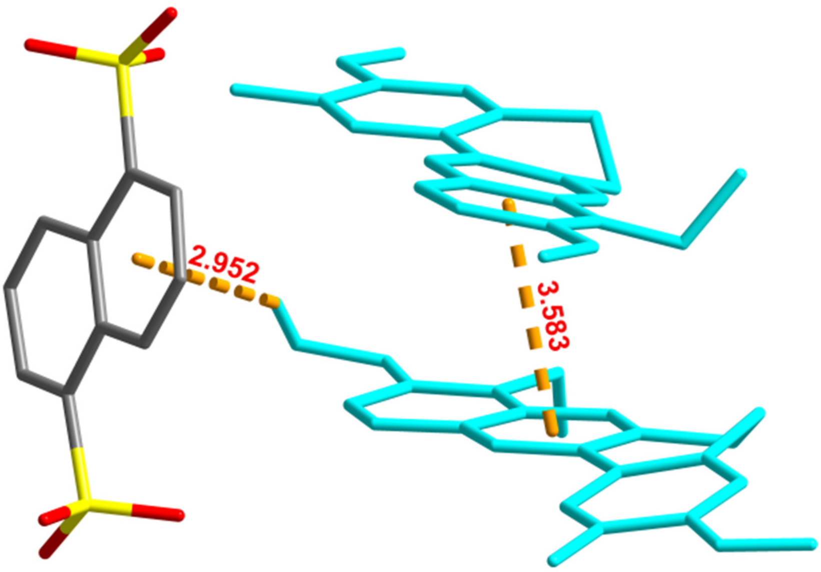

Figure S2d C–H···π interactions in **NDS⊃2**$_2$

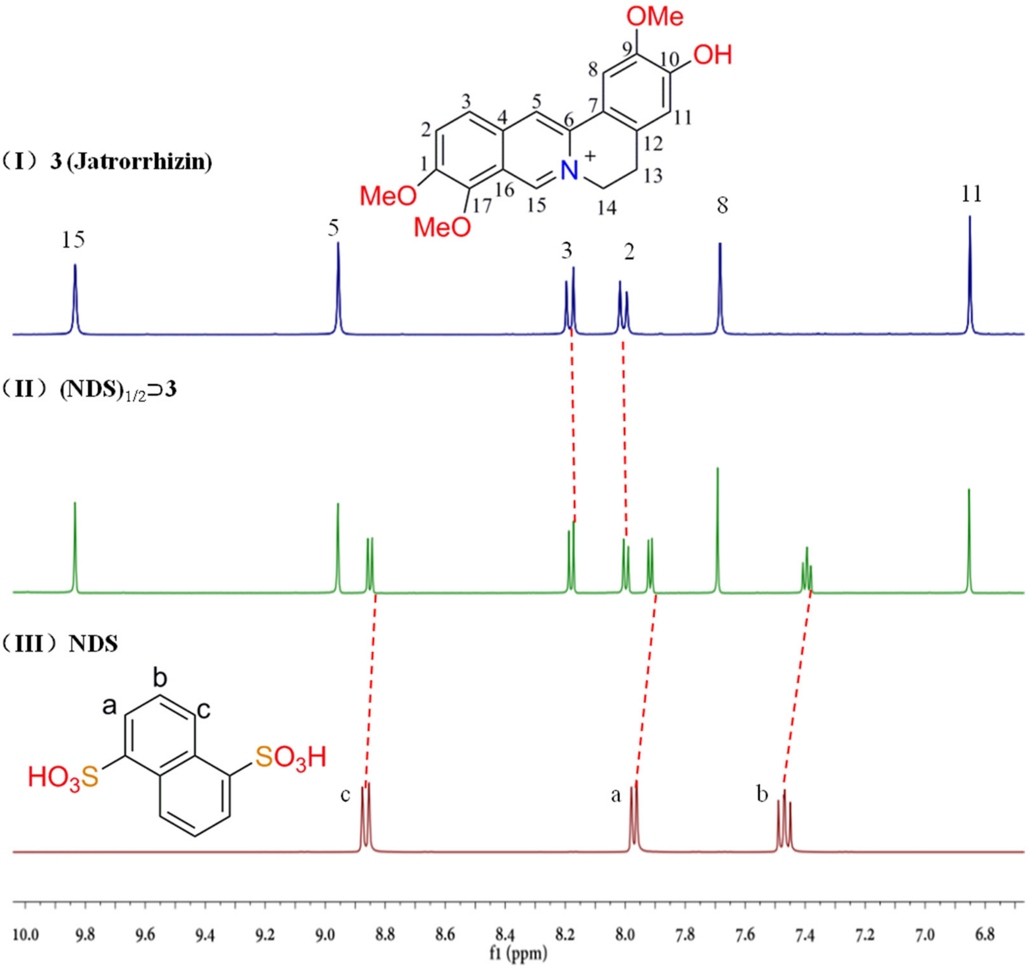

Figure S3a. ^{1}H-NMR of **3** (I), **(NDS)**$_{1/2}$⊃**3** (II) and NDS (III) (600 MHz, DMSO)

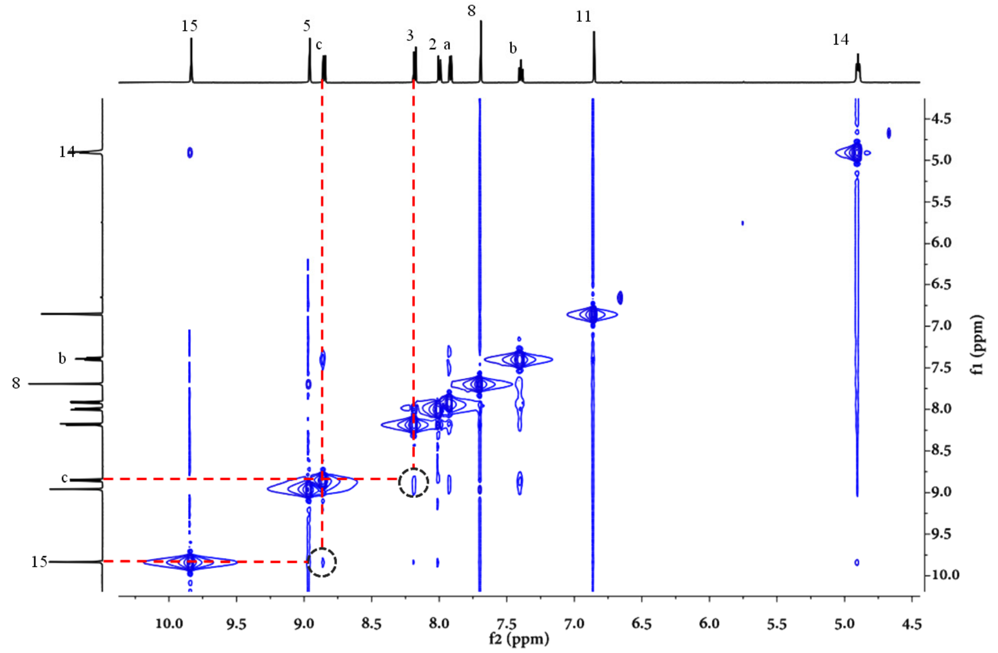

Figure S3b. NOESY spectra of **(NDS)**$_{1/2}$⊃**3** (600 MHz, DMSO) showing the correlations between Hc of NDS and H-3 of **3**, and between Hc of NDS and H-15 of **3**

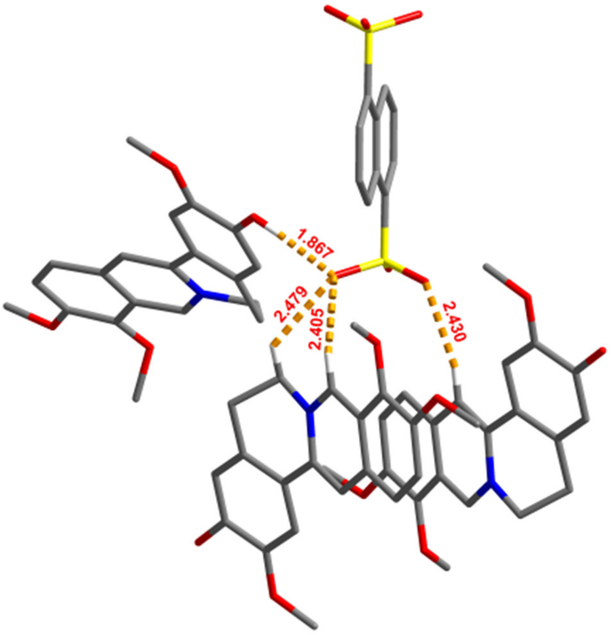

Figure S3c C–H···O interactions in **(NDS)**$_{1/2}$⊃**3**

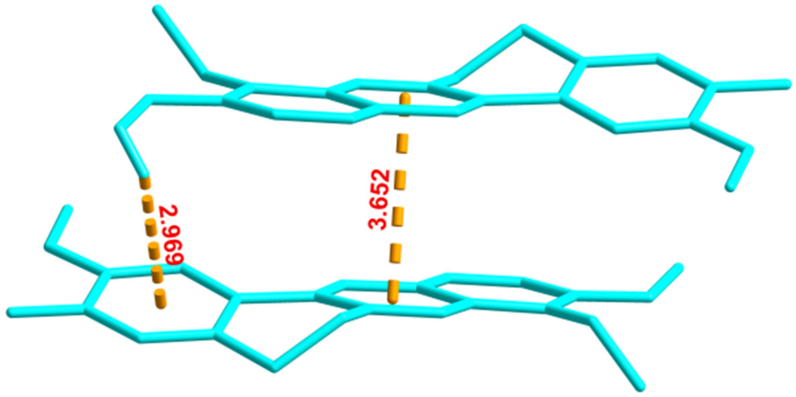

Figure S3d C–H···π interactions in **(NDS)**$_{1/2}$⊃**3**

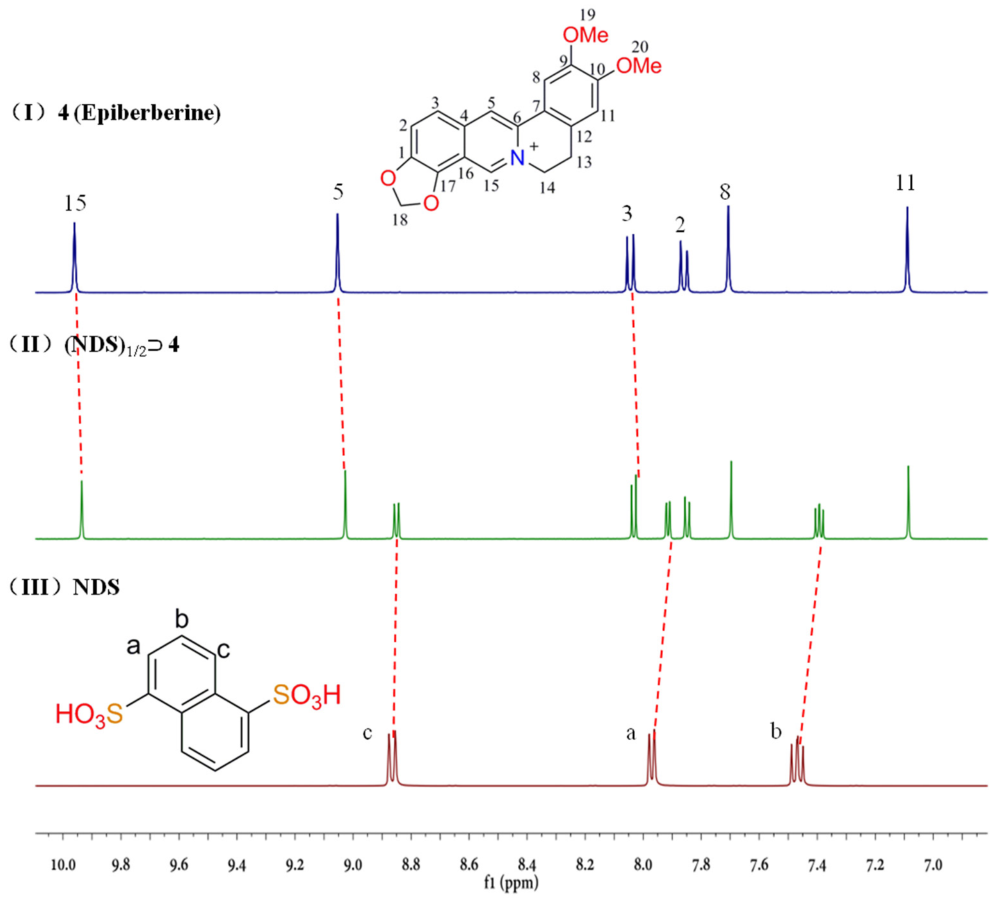

Figure S4a ^{1}H-NMR of **4** (I), **(NDS)**$_{1/2}$⊃**4** (II) and NDS (III) (600 MHz, DMSO)

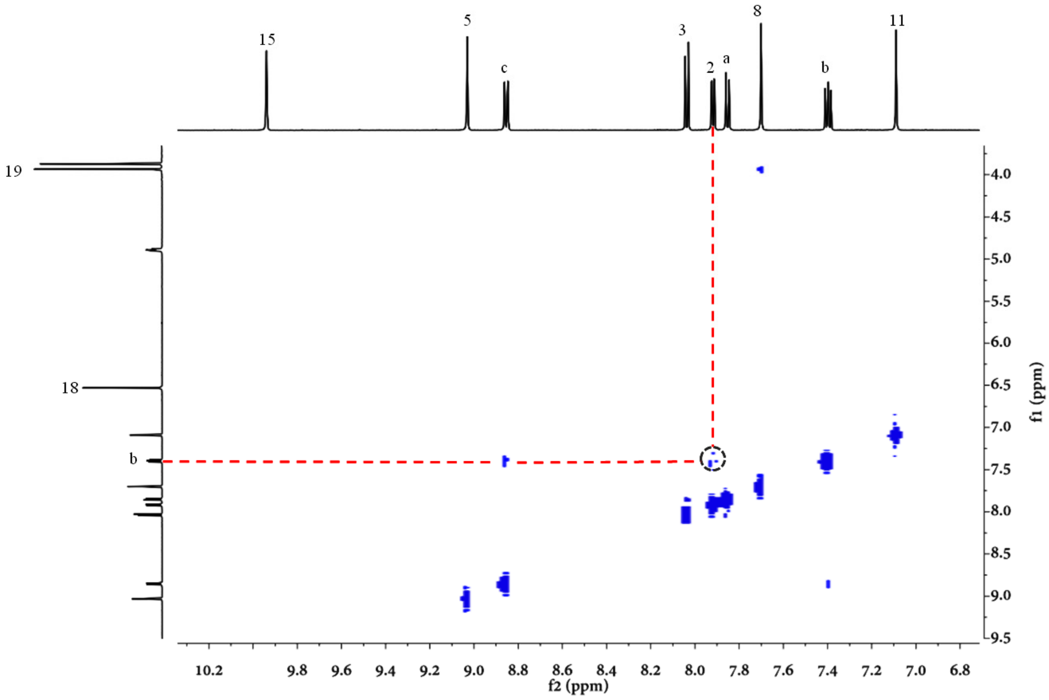

Figure S4b NOESY spectra of **(NDS)**$_{1/2}$**⊃4** (600 MHz, DMSO) showing the correlation between Hc of NDS and H-2 of **4**.

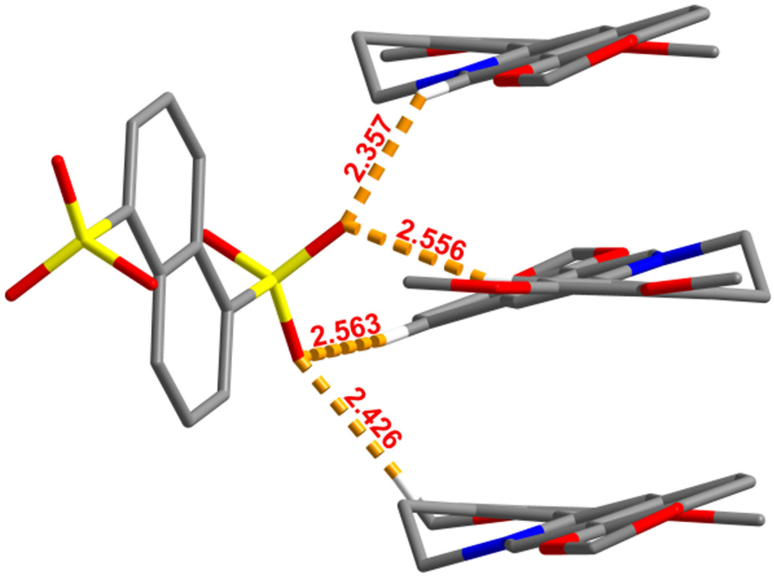

Figure S4c C−H···O interactions in **(NDS)**$_{1/2}$⊃**4**

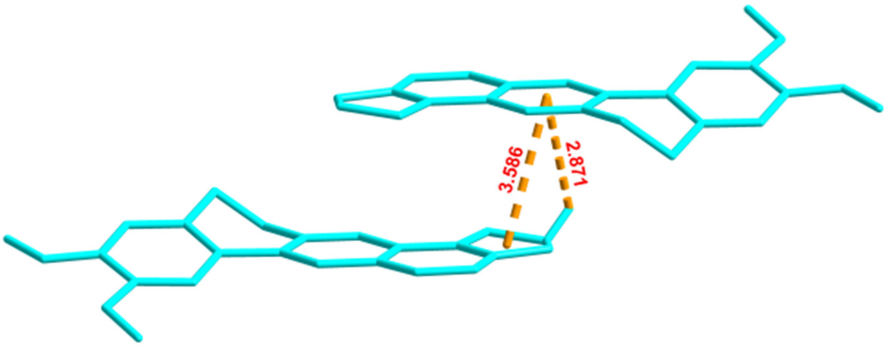

Figure S4d   C–H···π interactions in **(NDS)**$_{1/2}$⊃**4**

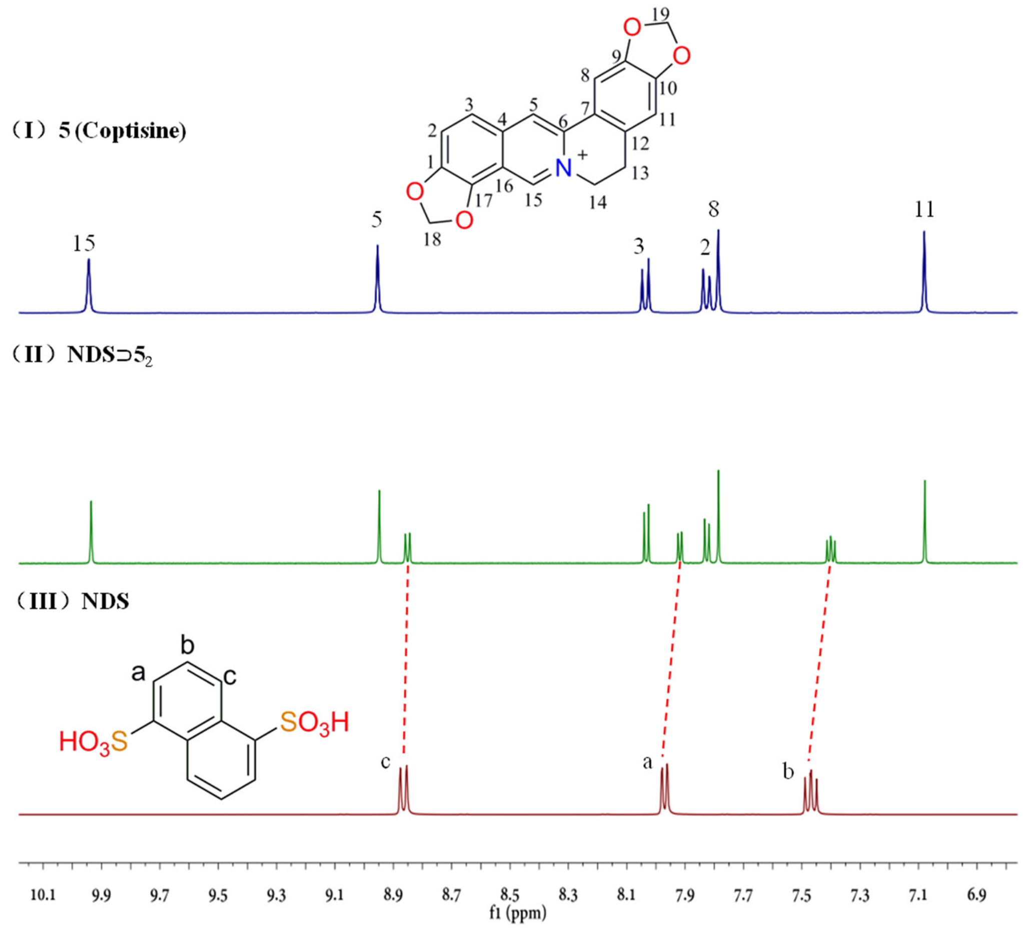

Figure S5a ^{1}H-NMR of **5** (I), **NDS⊃5**$_2$ (II) and NDS (III) (600 MHz, DMSO)

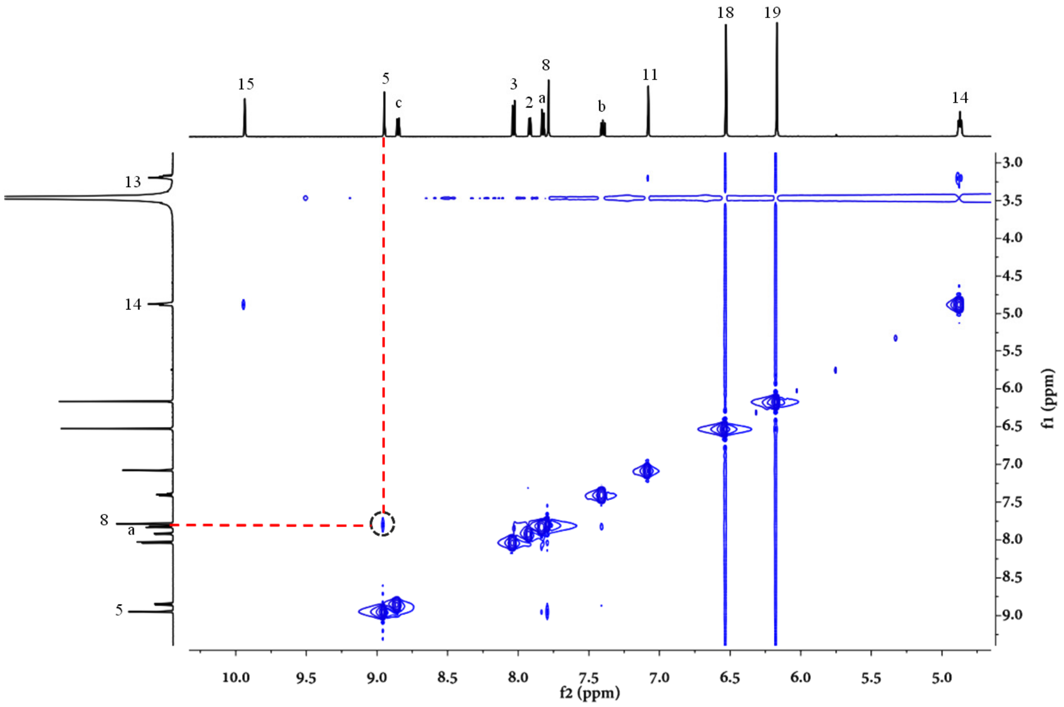

Figure S5b NOESY spectra of **NDS⊃5**2 (600 MHz, DMSO) showing the correlation between Ha of NDS and H-5 of **5**.

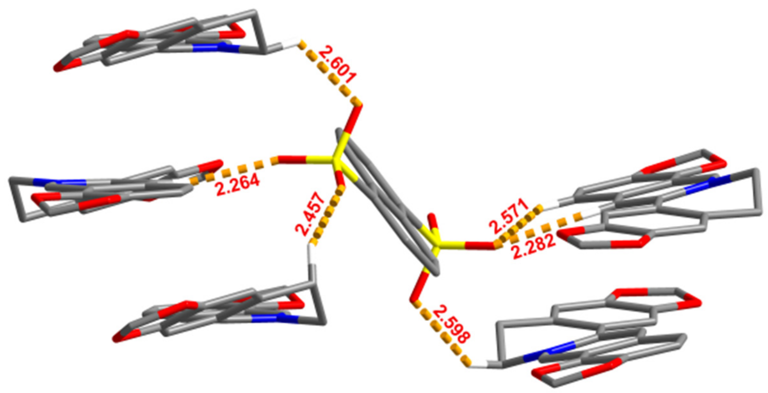

Figure S5c C–H···O interactions in **NDS⊃5**$_2$

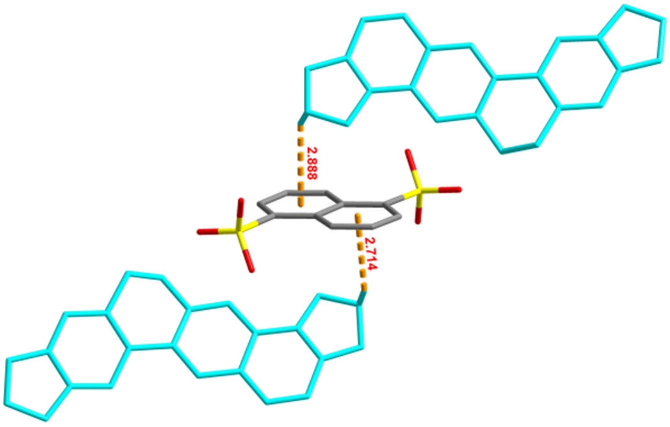

Figure S5d C–H···π interactions in **NDS⊃5**$_2$

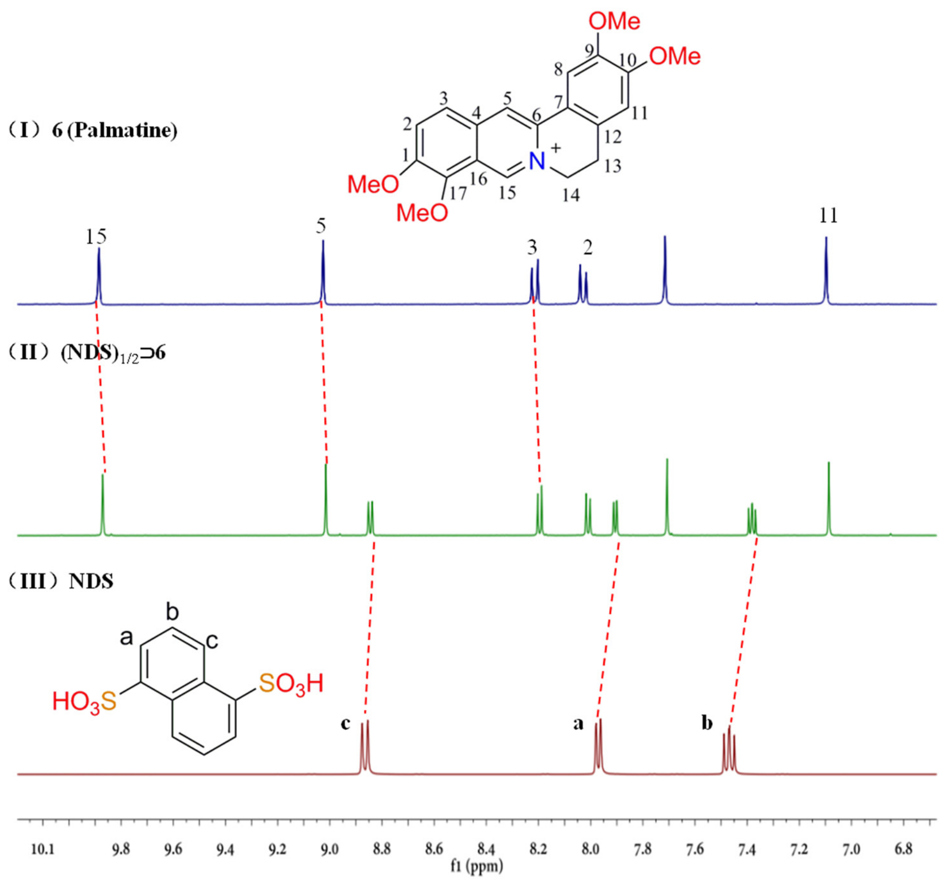

Figure S6a. ^{1}H-NMR of **6** (I), **(NDS)**$_{1/2}$⊃**6** (II) and NDS (III) (600 MHz, DMSO)

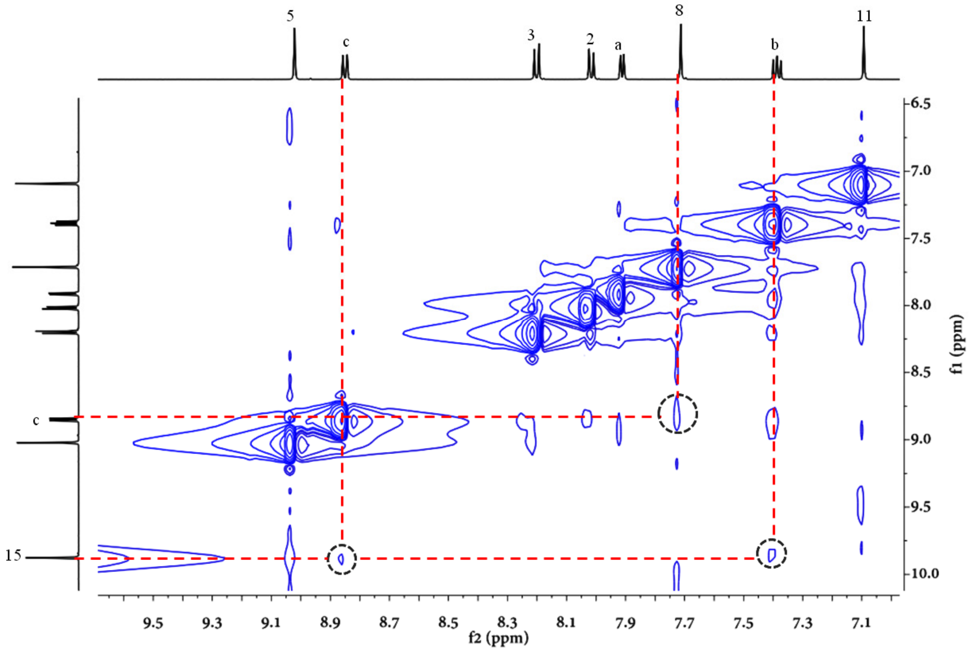

Figure S6b. NOESY spectra of **(NDS)**$_{1/2}$⊃**6** (600 MHz, DMSO) showing the correlations between Hc of NDS and H-8, H-15 of **6**, and between Hb of NDS and H-15 of **6**.

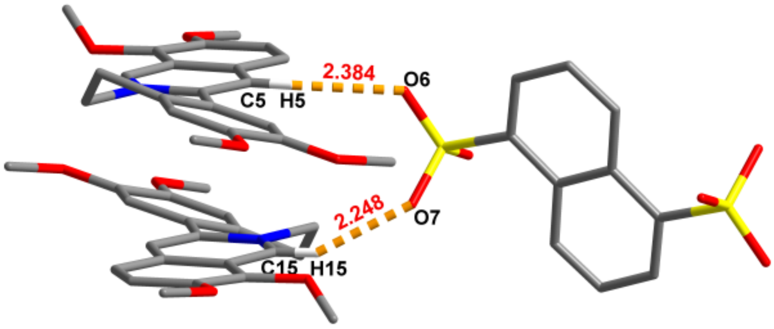

Figure S6c C–H···O interactions in **(NDS)**$_{1/2}$⊃**6**

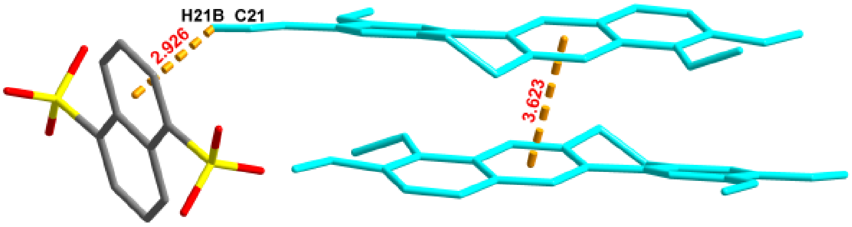

Figure S6d C–H···π interactions in **(NDS)**$_{1/2}$⊃**6**

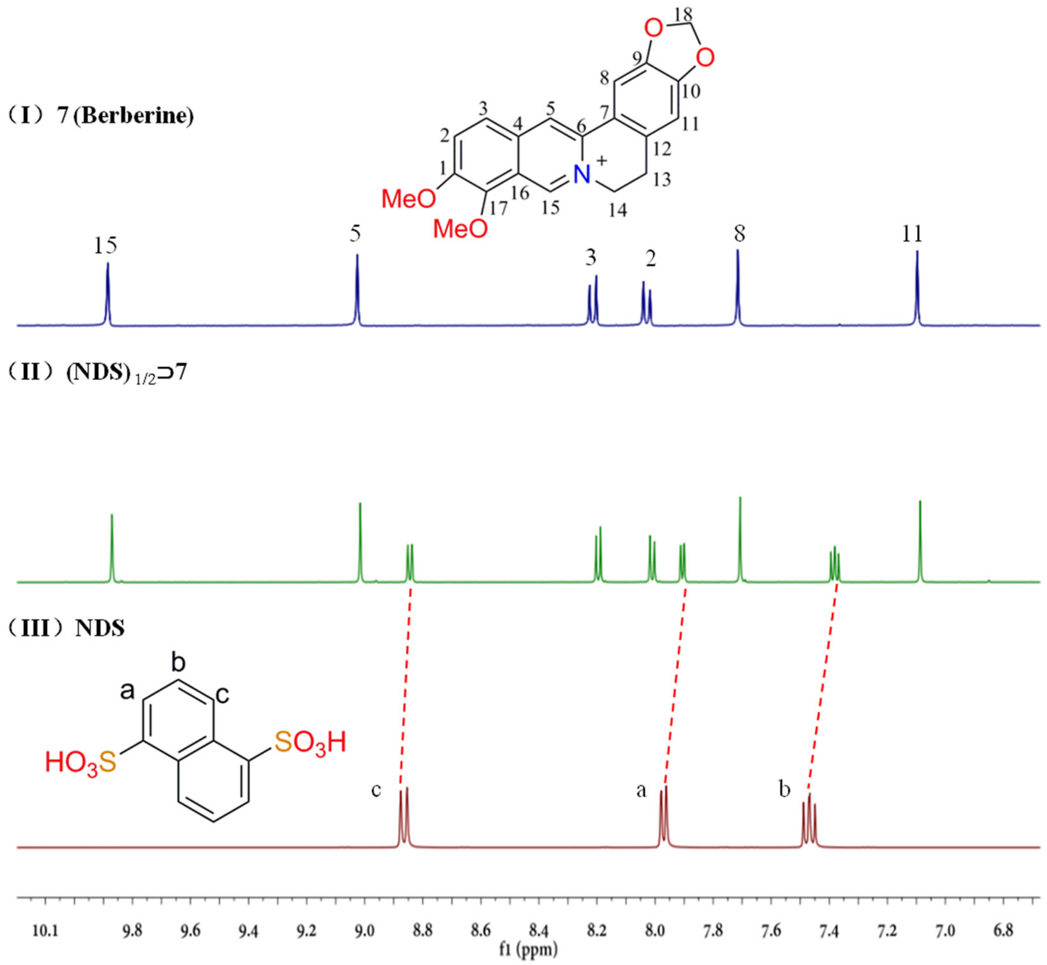

Figure S7a. ^{1}H-NMR of **7** (I), **(NDS)**$_{1/2}$⊃**7** (II) and NDS (III) (600 MHz, DMSO)

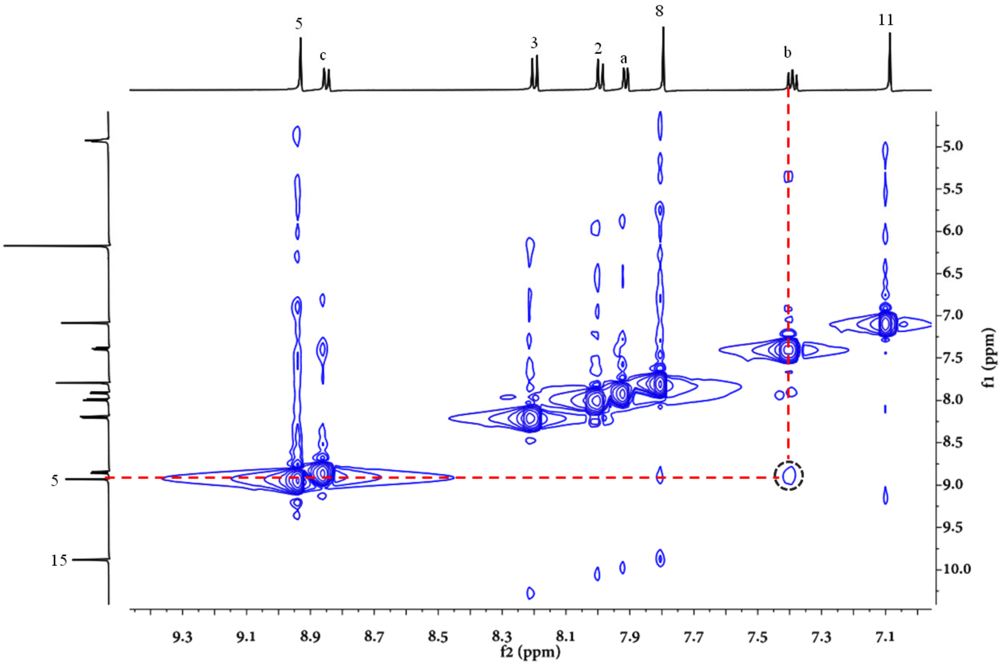

Figure S7b. NOESY spectra of **(NDS)**$_{1/2}$⊃**7** (600 MHz, DMSO) showing the correlation between Hb of NDS and H-5 of **7**.

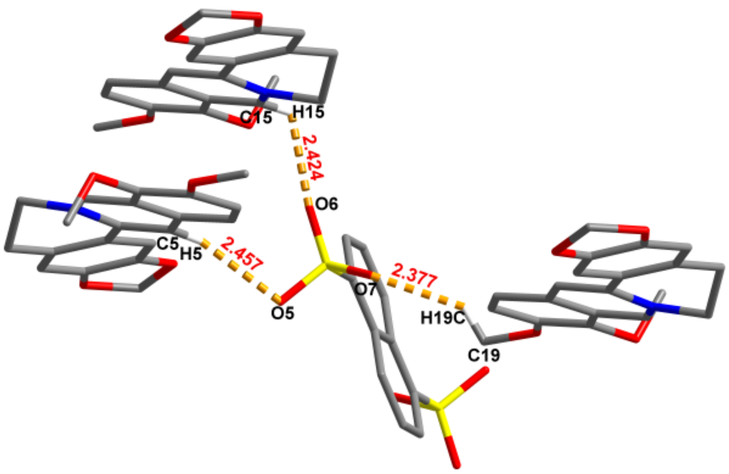

Figure S7c C–H···O interactions in **(NDS)**$_{1/2}$⊃**7**;

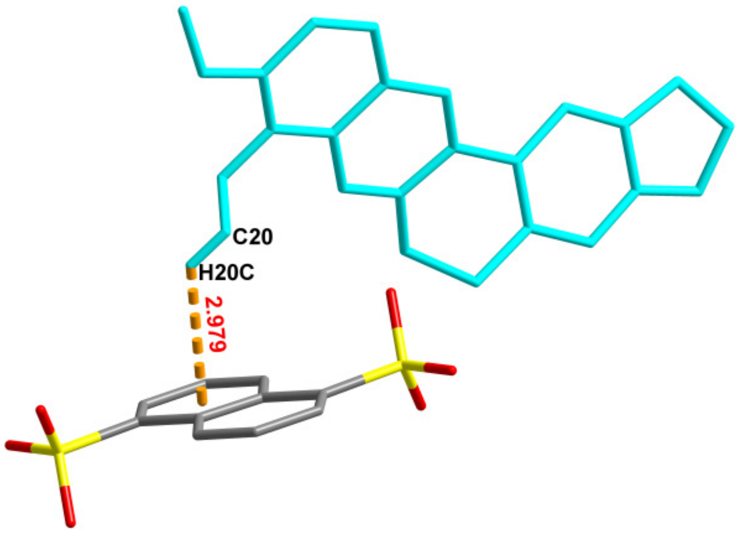

Figure S7d C−H···π interactions in **(NDS)**$_{1/2}$⊃**7**

5. Co-crystallization conditions

The co-crystallization conditions were shown in Table S1.

Table S1. Crystallization conditions

Co-crystals	solvent	Temperature	Time	Color, habit
**NDS⊃1**$_2$	methanol	20℃	2 min	Colorless, block
**NDS⊃2**$_2$	methanol	20℃	6 hours	Yellow, block
**(NDS)**$_{1/2}$**⊃3**	methanol	20℃	48 hours	Orange, needle
**(NDS)**$_{1/2}$**⊃4**	methanol	20℃	6 hours	Orange, block
**NDS⊃5**$_2$	methanol	50℃	48 hours	Yellow, needle
**(NDS)**$_{1/2}$**⊃6**	methanol	50℃	24 hours	Yellow, needle
**(NDS)**$_{1/2}$**⊃7**	methanol	50℃	24 hours	Yellow, needle

6. NDS decrease the required amounts of alkaloids to nanoscale

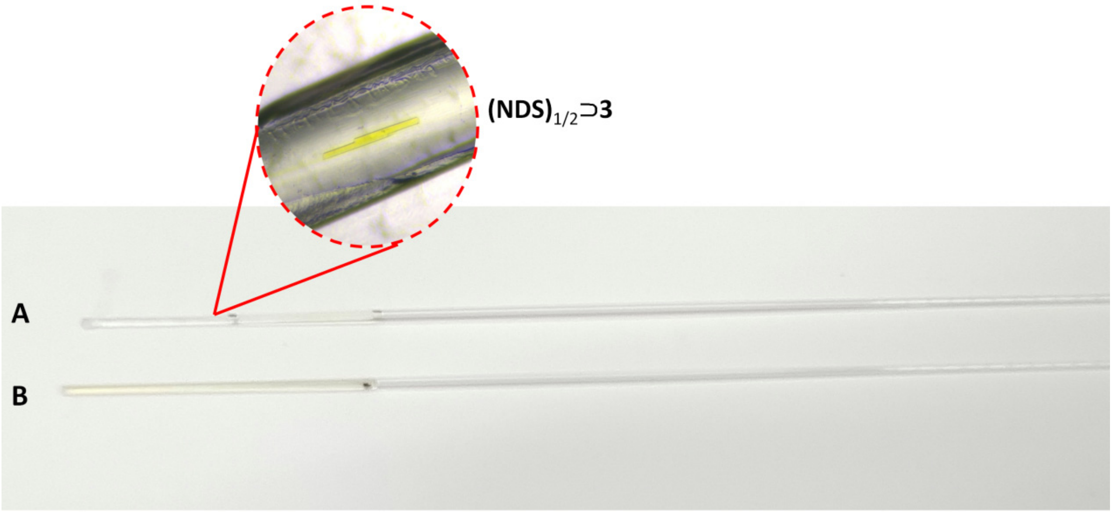

Figure S8 Nanoscale crystallization of **NDS** and **3**(jatrorrhizine). A: **3** (0.5μg/μL, 0.7μL) + **NDS** (0.5μg/μL, 0.7μL) (**3** placed on the upper layer of **NDS**); B: **3**(0.5μg/μL, 1.4μL). **(**the solvent is MeOH).

7. Interaction geometries in the co-crystal complexes

Table S2. Geometric parameters of the interactions in the co-crystals complexes

Co-crystals	Interactions	D−H (Å)	D···A (Å)	H···A (Å)	D-H···A (deg)	Symmetry code
	C_4A-H_4AB···O_6	0.970	2.4071	3.220(7)	141	1+x, 1+y, z
	C_5A-H_5AB···O_1	0.970	2.5739	3.503(5)	160	1+x, 1+y, -1+z
	C_8-H_8···O_3A	0.930	2.5921	3.504(6)	167	-1+x, -1+y, z
	C_5B-H_5BA···O_4	0.970	2.4976	3.313(6)	142	x, y, 1+z
**NDS⊃1**$_2$	C_5B-H_5BB···O_2A	0.970	2.3895	3.209(6)	142	x, y, z
	C_9A-H_9A···O_3	0.930	2.4636	3.364(7)	163	x, 1+y, z
	C_9B-H_9B···O_4	0.930	2.4948	3.412(7)	169	1+x, y, z
	C_{12}B-H_{12}D···O_1	0.960	2.4169	3.274(7)	149	x, y, z
	C_{13}A-H_{13}B···O_5	0.960	2.4824	3.347(7)	150	x, 1+y, z
	C_{13}B-H_{13}F···O_2	0.960	2.3301	3.121(7)	139	1+x, y, z
	O_3A-H_3A···O_1	0.820	1.9275	2.716(4)	161	x, y, z
	O_3B-H_3BA···O_2	0.820	1.9474	2.751(4)	166	x, y, z
	C_2B-H_2B···O_5	0.930	2.5395	3.394(5)	153	x, 1-y, 1/2+z
	C_{20}A-H_{20}B···O_4B	0.960	2.5427	3.297(6)	136	-1/2+x, 1/2-y, 1/2+z
**NDS⊃2**$_2$	C_{20}B-H_{20}D···O_4A	0.960	2.4128	3.247(5)	145	1/2+x, 1/2-y, -1/2+z
	C_{11}A-H_{11}A···O_4	0.930	2.4111	3.310(5)	163	1/2+x, 1/2-y, 1/2+z
	C_{14}B-H_{14}C···O_1A	0.970	2.5935	3.177(4)	119	-1+x, y, z
	C_{15}B-H_{15}B···O_3	0.930	2.3349	3.190(5)	153	-1/2+x, -1/2+y, z
	C_{19}B-H_{19}F···O_2A	0.960	2.5929	3.495(5)	157	-3/2+x, 1/2-y, 1/2+z
	O_3-H_3···O_6	0.820	1.8674	2.687(2)	177	x, y, z
	C_3-H_3A···O_7	0.930	2.4397	3.370(3)	179	-1/2+x,1/2-y, -1/2+z
	C_{15}–H_{15}···O_6	0.930	2.4047	3.172(3)	140	3/2-x,1/2+y,3/2-z
**(NDS)**$_{1/2}$**⊃3**	C_5 –H_5···O_5	0.930	2.4303	3.343(3)	167	-1/2+x,1/2-y, -1/2+z
	C_{13}–H_{13}A···O_7	0.970	2.5559	3.336(3)	137	3/2-x, -1/2+y, 3/2-z
	C_{14}-H_{14}B···O_6	0.970	2.4798	3.160(3)	127	3/2-x, 1/2+y, 3/2-z
	C_{20}-H_{20}B···O_1	0.960	2.5879	3.514(4)	162	1-x, 1-y, 1-z
	C_3-H_3···O_6	0.930	2.5626	3.430(3)	155	1-x, 1-y, 1-z
	C_8-H_8···O_5	0.930	2.5558	3.457(3)	163	1-x, 1-y, 1-z
**(NDS)**$_{1/2}$**⊃4**	C_{15}-H_{15}···O_5	0.930	2.3569	3.026(3)	129	x, y, z
	C_{18}-H_{18}A···O_4	0.970	2.4593	3.125(3)	126	1+x, 1+y, z
	C_{18}-H_{18}B···O_3	0.970	2.5683	3.353(3)	138	1-x, 1-y, 1-z
	C_{20}-H_{20}C···O_6	0.960	2.4256	3.334(4)	158	1-x, 1-y, 1-z
	C_2A-H_2A···O_4B	0.930	2.5388	3.450(4)	166	1-x, 1-y, 2-z
**NDS⊃5**$_2$	C_2B-H_2B···O_4A	0.930	2.5676	3.333(4)	140	1-x, -y, 1-z
	C_3A-H_3A···O_4	0.930	2.5715	3.349(4)	141	1-x, 1-y, 1-z

	$C_5A\text{-}H_5A\cdots O_4$	0.930	2.2826	3.142(4)	153	1-x, 1-y, 1-z
	$C_5B\text{-}H_5B\cdots O_1$	0.930	2.2636	3.119(4)	153	1-x, -y, 1-z
	$C_{13}B\text{-}H_{13}C\cdots O_2$	0.970	2.5462	3.353(4)	141	x, y, z
	$C_{14}A\text{-}H_{14}A\cdots O_4B$	0.970	2.5628	3.430(4)	149	x, y, z
	$C_{14}A\text{-}H_{14}B\cdots O_5$	0.970	2.5982	3.405(4)	141	1+x, y, 1+z
	$C_{14}B\text{-}H_{14}D\cdots O_3$	0.970	2.6005	3.267(4)	126	x, y, z
	$C_5\text{-}H_5\cdots O_6$	0.930	2.3988	3.259(3)	157	1-x, 1-y, 1-z
**(NDS)$_{1/2}$⊃6**	$C_{15}\text{-}H_{15}\cdots O_7$	0.930	2.2495	3.089(3)	150	x, y, z
	$C_{18}\text{–}H_{18}B\cdots O_3$	0.930	2.5884	3.339(3)	136	x, y, -1+z
	$C_{20}\text{–}H_{20}B\cdots O_1$	0.930	2.5084	3.293(3)	139	1-x, 1-y, 1-z
	$C_5\text{-}H_5\cdots O_5$ [c]	0.930	2.4570	3.302(4)	151	1-x, 1-y, 1-z
**(NDS)$_{1/2}$⊃7**	$C_{18}\text{–}H_{18}A\cdots O_1$	0.970	2.5723	3.088(5)	113	x, 1+y, -1+z
	$C_{18}\text{–}H_{18}B\cdots O_3$	0.970	2.5194	3.323(5)	140	-x, 2-y, -z
	$C_{19}\text{-}H_{19}C\cdots O_7$	0.960	2.3768	3.166(5)	139	x, -1+y, z

8. Single-crystal X-ray diffraction analysis

All single-crystal measurements were performed on a Rigaku Oxford diffractometer using CuKα (λ = 1.54056 Å) radiation. The diffraction data were collected in the ω-scanning mode. The structures were solved by direct methods (SHELXTL-2014) and refined by full-matrix least-squares on F^2. In the structure refinements, non-H atoms were refined anisotropically. H atoms bonded to carbons were placed on the geometrically ideal positions by the 'ride on' method. H atoms bonded to oxygen were located by the difference Fourier method and were included in the calculation of structure factors with isotropic temperature factors. No significant disorders were observed in the host, guest and solvent molecules, and thus no restrain or constrain instructions was used in the refinements.

Crystallographic data for the structures reported in this paper have been deposited with the Cambridge Crystallographic Data Centre. The CCDC numbers are shown in Table 3. Copies of the data can be obtained, free of charge, on application to CCDC, 12 Union Road, Cambridge CB2 1EZ, U.K. [fax: (+44)1223-336033; e-mail: deposit@ccdc.cam.ac.uk].

9. Electrostatic potential surfaces

The theoretical calculations were performed using the B3LYP time-dependent density functional theory method at the 6-31G(d) level in the Gaussian 09 program. The electrostatic potential surfaces (ESP) of computed species were generated with Gaussview 6.0.

Cartesian coordinates (Å) for the optimized geometries of NDS

C	-1.75950000	1.68510000	0.10990000
C	-1.76600000	0.34110000	0.09940000
C	-0.59210000	-0.33500000	0.09950000
C	0.58940000	0.33500000	0.09560000
C	0.55140000	1.68230000	0.10450000
C	-0.60430000	2.35770000	0.11410000
C	-0.55410000	-1.68230000	0.11020000
C	0.60160000	-2.35780000	0.11070000
C	1.75670000	-1.68510000	0.09890000
C	1.76330000	-0.34110000	0.08860000
S	-3.39030000	-0.40930000	0.07220000
O	-4.50020000	0.39920000	-0.44280000
O	-3.64260000	-1.47960000	1.04470000
O	-3.58710000	-1.63930000	-1.04340000
S	3.38760000	0.40930000	0.06960000
O	4.50020000	-0.39900000	-0.44000000
O	3.63500000	1.47950000	1.04360000
O	3.59030000	1.63960000	-1.04470000
H	-2.69490000	2.26990000	0.11500000

H	1.45460000	2.30830000	0.11040000
H	-0.60700000	3.46110000	0.12400000
H	-1.45740000	-2.30820000	0.12470000
H	0.60440000	-3.46110000	0.12260000
H	2.69220000	-2.27000000	0.10300000
H	-3.92700000	-2.40040000	-0.61020000
H	3.92800000	2.40050000	-0.60950000

Cartesian coordinates (Å) for the optimized geometries of 1

C	-2.52950000	-0.88690000	-0.28600000
C	-2.51230000	-2.21590000	-0.10780000
C	-1.37360000	-2.83740000	0.20760000
C	-0.23520000	-2.13960000	0.34340000
C	-0.20610000	-0.79530000	0.19880000
C	-1.36870000	-0.20290000	-0.18120000
C	1.03320000	-2.89760000	0.63950000
C	2.07970000	-2.27570000	-0.28790000
C	2.12820000	-0.80510000	0.08230000
C	0.97870000	-0.13870000	0.36190000
N	3.42310000	-2.99640000	-0.17120000
C	4.44250000	-2.19370000	-0.96030000
C	4.61160000	-0.82480000	-0.30190000
C	3.31050000	-0.16590000	0.08760000
C	3.34820000	1.11460000	0.47230000
C	2.26230000	1.78450000	0.88670000

C	1.07350000	1.14100000	0.81340000
O	-1.43500000	1.10800000	-0.54650000
O	-0.01020000	1.78610000	1.32470000
C	3.34470000	-4.37440000	-0.81840000
C	3.87600000	-3.18330000	1.26880000
O	-3.67320000	-0.26460000	-0.72150000
C	-4.54630000	0.11240000	0.31840000
O	2.47100000	3.08340000	1.30130000
C	1.45520000	4.05060000	1.17780000
H	-3.43340000	-2.80410000	-0.25530000
H	-1.38220000	-3.93350000	0.32540000
H	1.26770000	-2.75940000	1.71980000
H	0.90830000	-3.98500000	0.44530000
H	1.73770000	-2.33320000	-1.35110000
H	5.42840000	-2.71390000	-1.00230000
H	4.09510000	-2.06440000	-2.01320000
H	5.15840000	-0.16270000	-1.01610000
H	5.25870000	-0.90330000	0.60310000
H	4.32370000	1.63060000	0.49180000
H	-2.25340000	1.29510000	-1.03050000
H	-0.75890000	1.17910000	1.41810000
H	4.33830000	-4.87760000	-0.80850000
H	2.64260000	-5.05240000	-0.28710000
H	3.01390000	-4.30810000	-1.87960000
H	3.83810000	-2.23760000	1.85200000

H	3.24430000	-3.92460000	1.80630000
H	4.91880000	-3.57190000	1.30850000
H	-5.42840000	0.61670000	-0.13540000
H	-4.88780000	-0.78790000	0.87640000
H	-4.03320000	0.82210000	1.00560000
H	1.93290000	5.05240000	1.26540000
H	0.97250000	3.98410000	0.17750000
H	0.72670000	3.96870000	2.01320000
